# SDF-1 induces directional chemotaxis of BMSCs at the intervertebral fusion site and promotes osteogenic differentiation by regulating Wnt/β-catenin in the bone marrow chimera spinal intervertebral fusion mouse model

**DOI:** 10.55730/1300-0152.2638

**Published:** 2022-12-21

**Authors:** Qiwen ZHANG, Ning LIANG, Bin HE, Siyou WU, Depeng WEN, Xiaoyong TANG, Xiongcheng SHEN

**Affiliations:** 1 People’s Hospital of Honghuagang District, Guizhou, China; 2 The Third Affiliated Hospital of Zunyi Medical University (Zunyi first people’s Hospital), Guizhou, China

**Keywords:** Spinal interbody fusion, bone marrow mesenchymal stem cells (BMSCs), stromal cell-derived factor (SDF-1), Wnt/β-catenin signaling pathway, osteogenic differentiation, chemotaxis

## Abstract

Clinical observations show that the current spinal fusion with internal fixation has a nonfusion rate of 5%–35%; however, methods to promote spinal fusion are limited. This study aimed to investigate the role of SDF-1-induced directional chemotaxis of BMSCs in bone marrow chimera spinal intervertebral fusion mouse model. BMSCs were isolated from bone marrow and identified by detecting CD44/CD34 positive cells. BMSCs (GFP-BMSCs) were labeled with GFP for tracking in vivo. Mice were inoculated with GFP-BMSCs to construct bone marrow chimera spinal intervertebral fusion model, which were divided into BM-SIF model, BM-SIF+SDF-1, BM-SIF+SDF-1-Anta group. The callus area of intervertebral fusion site was detected by radiology. HE staining was used to detect trabeculae formation. Expressions of osteogenic molecules and fibroblast markers were detected by RT-PCR and Western blotting. GFP-BMSCs showed obvious osteogenic and adipogenic differentiation ability, according to oil-red O and alizarin-red staining. Bone marrow chimera spinal intervertebral fusion mouse model was successfully established, with efficient localization of GFP-BMSCs at intervertebral fusion site. SDF-1 significantly promoted bone trabeculae formation in callus at intervertebral fusion site. SDF-1 significantly increased osteogenic molecules transcription/expression in callus at intervertebral bone graft fusion site of mice; however, SDF-1-Anta (AMD3100) significantly decreased osteogenic molecules transcrition/expression, compared to those of mice from the BM-SIF model group (p < 0.05). SDF-1 markedly induced and SDF-1-Anta significantly decreased fibroblast proliferations in the callus at the intervertebral fusion site of mice, compared to those of mice from the BM-SIF model group (p < 0.05). SDF-1 enhanced expression of Wnt10b and β-catenin in callus at intervertebral fusion site of mice compared to mice of the BM-SIF model group (p < 0.05). In conclusion, SDF-1 induced directional chemotaxis of BMSCs to the intervertebral fusion site and promoted osteogenic differentiation in bone marrow chimera spinal intervertebral fusion mice by regulating Wnt/β-catenin pathway and modulating the proliferation of BMSCs.

## 1. Introduction

The spinal interbody fusion technique is a conventional treatment for spinal degeneration, tumor, trauma, and deformity (Jeremy Goh et al., 2019; Shah et al., 2019). Spinal interbody fusion can make the bone graft fusion segment achieve solid bone fixation, and it can also expand and maintain the normal height of interbody fusion. However, the problem that spine surgeons face in clinical practice is fusion failure. A large number of clinical observations have shown that the current spinal fusion with internal fixation has a nonfusion rate of about 5%–35%. Currently, clinical methods to promote spinal fusion are limited only to improve the construction of the bone graft bed and the application of spinal internal fixation instruments (Tropiano et al., 2017). Because the mechanism of bone graft fusion in spinal fusion has not been fully clarified, it brings great confusion to spinal surgeons in clinical work. Therefore, how to effectively improve the fusion rate of spinal fusion at the molecular level has become a research hotspot in the field of spinal surgery.

Human bone marrow contains a large number of bone marrow mesenchymal stem cells (BMSCs) (Jiang et al., 2021). These BMSCs can participate in immune rejection and other advantages and can continue to migrate to the wound site for a period of time. However, in the absence of other factors, the number of BMSCs migrating to the wound site to participate in tissue repair is very small (Liu et al., 2021). This short-term, low-dose recruitment of BMSCs obviously cannot meet the requirements for bone repair after serious fractures or most bone defects.

Stromal cell-derived factor (SDF-1) belongs to class A chemokine, also known as CXCR12, which is a cytokine with relatively small molecular weight (Wang et al., 2022). Previous studies have confirmed that SDF-1 can recruit cells and factors necessary for bone healing to the site of bone injury and participate in bone tissue repair (Cai et al., 2021). The SDF-1 molecule can also promote BMSCs migration (Zhang and He, 2019). In the field of orthopedics, the former study (Qin et al., 2021) on osteoarthritis (OA) has shown that the angiogenesis of the OA articular cartilage is related to osteogenesis and the SDF-1/CXCR4 signaling pathway. There are many similarities between the physiological microenvironment of the knee joint and spine in vivo (low nutrition, hypoxia, low blood supply, high stress) (Yu et al., 2019). Therefore, our team speculated that osteogenic differentiation chemotaxis of BMSCs mediated by the SDF-1/CXCR4 signaling pathway also plays an important role in intervertebral fusion.

Wnt/β-catenin signaling pathway plays a key role in the growth, development, and differentiation of embryonic stem cells, bone production, and insulin glargine metabolism (Wang et al., 2021). The classical Wnt signaling pathway can regulate osteogenic differentiation, bone matrix formation, and mineralization, and affect osteoclast formation and bone resorption (Khalifé et al., 2021). Some studies (Siar et al., 2012; Huang et al., 2017) have shown that Wnt10b is a canonical/wnt ligand expression in bone formation, and its effect on BMSCs is unique. When the Wnt10b gene is removed, the number of BMSCs will be reduced and osteogenic ability will also be reduced (Jing et al., 2022). Therefore, Wnt/β-catenin signaling pathway is closely related to bone formation. The Wnt pathway is a key signal pathway that regulates the differentiation of BMSCs into osteoblasts, and it is involved mainly in the formation, differentiation, and proliferation of BMSCs into osteoblasts. However, it has not been reported whether SDF-1 also regulates new bone formation through this signal pathway in bone grafting and fusion of the spinal intervertebral space.

In summary, this study successfully constructed a spinal intervertebral fusion mouse model of the bone marrow chimera. BMSCs in the mouse model carry a green fluorescent protein (GFP), which can trace and observe the regulatory effect of SDF-1 on the directional migration of BMSCs in the mouse model. In this study, the osteogenic ability of the constructed mouse model was detected and the signal pathway was verified. This study elucidated the regulatory mechanism of SDF-1 that induces the directional chemotaxis of BMSCs in the spinal intervertebral fusion process through in vivo and in vitro experiments. The development of this study will provide a new idea for spinal interbody fusion.

## 2. Materials and methods

### 2.1. Animals

Specific pathogen-free (SPF) C57BL/6 mice aging 6–8 weeks and weighing 25 g (Certificate No. SCXK(Chuan)-2020-030) were purchased from Chengdu Dossy Exp. Animal Co. Ltd. The mice were kept at a temperature of 23–25 °C in an environment of a 12 h/12 h cycle of light/dark. During the whole experimental process, the mice had free access to the commercial diet and water. All experiments were carried out after an acclimation time of 1 week.

The protocol for the use of mice in this study has been approved by the Animal Care and Use Committee of the People’s Hospital of Honghuagang District, Zunyi, China. All experiments on mice were carried out according to the Guide for the Care and Use of Laboratory Animals of People’s Hospital of Honghuagang District.

### 2.2. Establishment of bone marrow chimera mouse model

BMSCs were isolated from the bone marrow of SPF C57BL/6 mice. Briefly, mice’ femur and tibia were separated, and bone marrow cells were washed with DMEM and made into a cell suspension. The BMSCs were obtained from bone marrow cell suspension by conventional centrifugation. The isolated cells above were cultured and the third generation cells (P3) were selected for identification. BMSCs were identified by detecting positively expressed CD44-FITC cells (CD44^+^) and negatively expressed CD34-FITC cells (CD34^−^) with flow cytometry, based on the report from a previous study (You et al., 2013). The GFP containing fluorescent lentivirus vector (plvx-mcmv-zsGreen1 vector) was mixed with psPAX2 and pMD2. G for virus packaging. BMSCs expressing GFP were then infected (transfected) with the packaged plvx-mcmv-zsGreen1 vector to generate the GFP-expressing BMSCs (GFP-BMSCs cells). The expression of GFP in BMSCs was identified by confocal microscopy and immunohistochemical assay (by staining with anti-GFP antibody). The osteogenic and adipogenic differentiation ability of the BMSCs was identified by oil-red O staining and alizarin-red staining, respectively. C57BL/6 mice were anesthetized by intraperitoneal injection (7% chloral hydrate) and exposed to X-ray irradiation in a prone position (6 Gy/time, twice and 3 min/time). About 4 h after X-ray irradiation, BMSCs infected with lentivirus vector expressing GFP (1 × 10^6^ GFP-BMSCs cells) were added into the cell suspension (1: 1000). Then a total of 0.3 mL of GFP-BMSCs cell suspensions were injected into the caudal vein of mice, assigned as the experimental group (GFP-BMSCs group, n = 66), while the same volume of cell-free medium was injected into the caudal vein of mice, as the control group (Control group, n = 12). At the 1st and 5th week after GFP-BMSCs transplantation, whole bone marrow cells from mice were taken and GFP expression was identified by flow cytometry. The evaluation/surveillance of the animal welfare of the mice was performed through the experiment and they were OK. Flow cytometry was used to detect whether BMSCs in mice in the above two groups carried and stably expressed GFP fluorescence for later observation of tracing.

### 2.3. Establishment of the spinal intervertebral fusion mouse model of the bone marrow chimera and grouping of trials

The bone marrow chimera model established above was used for interbody fusion surgery. After adaptive feeding of bone marrow chimeric mice for 1 week, all mice’s indexes were normal. The bone marrow chimera spinal intervertebral fusion mouse model was established as described by Zhang et al. (2016), with a few modifications. The spinal intervertebral fusion model was established after intraperitoneal injection of 7% chloral hydrate. A total of 36 bone marrow chimeric mice were divided into the BM-SIF model group (n = 12), the BM-SIF+SDF-1 group (n = 12), the BM-SIF+SDF-1-Anta group (n = 12), and each group was divided into three time points for sampling and detection (7, 18, 28 days). The mice sat prone on the operating table and the back operation area was prepared and disinfected. The skin was cut through the posterior median incision and the fascia was cut 3 mm on both sides of the spinous process. The spine was exposed, tissues were separated to expose the intervertebral disc of the lumbar spine, and the intervertebral disc was removed. The upper and lower vertebral bodies were ground with a dental drill to expose the cartilage of the endplate, and the preparation of the bone graft bed was completed. Autologous bones were prepared into granular bone and filled in the grinding mouth of the bone grafting bed. Meanwhile, 0.1 mL of ECM solution containing 100 ng/mL of SDF-1 and containing 2 ng/mL of the SDF-1 (CXCR4) antagonist (AMD3100, SDF-1-Anta) was injected into the bone grafting bed of mice in the SDF-1 group and the BM-SIF+SDF-1-Anta group, respectively. The same volume of ECM solution was injected into the bone grafting bed of mice in the BM-SDF-1 group. After the operation, the incision was closed layer by layer with 4-0 absorbable suture. The mice were then kept in separate cages and buprenorphine was injected every 12 h to relieve pain, lasting 2 days. Penicillin sodium was injected intramuscularly for 3 days after the operation and the suture was removed 10 days after the operation.

### 2.4. Measurement of the area of the intervertebral callus

The mice in each group were detected by radiology on the 7th, 18th, and 28th days after the operation. Measurement of the area of the intervertebral callus was carried out as a description of a previous study (Xiao et al., 2018) with some modifications. Briefly, mice were killed at the corresponding time points mentioned above and intervertebral bone tissue was taken at the spinal fusion site. After washing with PBS, bone tissue was fixed with 4% paraformaldehyde for 24 h, and radiological examination was performed on a micro-CT machine as instructed by the manufacturer’s protocol (Viva CT 40, SCANCO Medical, Switzerland). The micro-CT scanner was set at a voltage of 40 kV, a current of 250 μA, and a resolution of 9 μm/pixel to measure intervertebral bone tissue. Images of bone tissues were reconstructed with NRecon v1.6 and the area of the intervertebral callus at the fusion site was analyzed with the micro-CT-associated CTAn v1.9 software. Finally, the callus area was calculated as described by a previously published study (Zhou et al., 2009), according to the above micro-CT reconstruction and scan results.

### 2.5. Detection of BMSCs in the callus of the intervertebral bone graft fusion site

Mice were taken at the corresponding time points (7th, 18th, and 28th days after operation) and the fusion site of the intervertebral bone graft samples of mice was used after radiological examination to detect BMSCs. Intervertebral bone graft fusion tissues were fixed with 4% paraformaldehyde, embedded in paraffin and cut into tissue sections with a thickness of 5 μm. After the tissue sections were soaked in PBS for 5 min, DAPI was incubated in the dark for 15 min for nuclear staining. The tissue sections were washed with PBS for 5 min and 4 times to remove excess DAPI. The absorbent paper was used to absorb the residual PBS in the tissue sections, and the tissue sections were sealed with an antifluorescence quenching agent. Finally, the BMSCs in bone tissue from the intervertebral bone graft fusion site of mice from each group were observed using a laser confocal microscope.

### 2.6. Histological evaluation

The above tissue sections of the intervertebral bone graft fusion site were prepared as described. Tissue sections were stained with hematoxylin and eosin (HE) staining, with 5 min of hematoxylin staining and 20 s of eosin staining. All tissue sections were evaluated under a light microscope and the images were photographed.

### 2.7. Real-time PCR (RT-qPCR) assay

Total RNAs in callus tissues of the intervertebral bone graft fusion site of mice were extracted using RNAiso plus 9109 following the manufacturer’s protocol (Takara, Tokyo, Japan). Complementary DNA (cDNA) was synthesized using 2 μg of total RNA as template, with a Hifair II 1st Strand cDNA synthesis kit (Cat. No. 11121ES60) as described by the manufacturer’s protocol (Yeasen Biotech., Shanghai, China). Target gene transcriptions, including α smooth muscle actin (α-SMA), collagen-1 (Col-1), osteocalcin (OCN), osteopontin (OPN), and bone sialoprotein (BSP), were amplified using a Hieff UNICON Universal Blue Qpcr SYBR Green Master Mix (Cat. No. 11184ES03, Yeasen Biotech., Shanghai, China) and determined using an AB Step One plus Real-Time PCR System (Applied Biosystems, Foster city, CA, USA). The reaction conditions of the RT-PCR assay were listed as follows: 95 °C for 2 min, and 40 cycles involving 95 °C for 10 s and 60 °C for 30 s. Transcriptions of the above targeting genes were calculated according to the description of a previously published 2^−^^ΔΔ^^Ct^ method, by normalizing to the housekeeping gene GAPDH (Livak and Schmittgen, 2001). The RT-PCR primer sequences are shown in Table.

### 2.8. Western blot assay

The callus tissues of the intervertebral bone graft fusion site of mice were lysed using RIPA lysis buffer (Cat. No. P0013, Beyotime Biotech., Shanghai, China, 100 mg tissues/1ml RIPA), the obtained lysates were centrifuged at 10000 × *g* for 10 min and the remaining supernatants were used for the following Western blot assay. The concentration of lysate (protein) was measured with a BCA protein assay kit (Cat. No. P0012, Beyotime Biotech.). Subsequently, approximately 20 μg of protein was denatured on 10% SDS-PAGE gels and electrotransferred onto PVDF membranes (Cat. No. FFP26, Beyotime Biotech.). The PVDF membranes were blocked with 5% nonfat milk for 1 h at room temperature and incubated with primary antibodies (including rabbit antimouse β-catenin antibody (Cat. No. ET1601-5, HuaBio., Zhejiang, China), antimouse Wnt10b antibody (Cat. No. DF9038, Affinity Biosciences Ltd.), antimouse BSP antibody (Cat. No. DF7738, Affinity Biosciences), antimouse OCN antibody (Cat. No. DF12303, Affinity Biosciences), antimouse OPN antibody (Cat No. AF0227, Affinity Biosciences) and antimouse β-actin antibody (Cat. No. ET1701-80, HuaBio.)) at 4 °C overnight. After washing with PBST solution, PVDF membranes were incubated with horseradish peroxidase (HRP) conjugated goat antirabbit IgG (Cat. No. A0208, Beyotime Biotech.) at room temperature for 1 h. The PVDF membranes were then treated with the BeyoECL Moon kit (Cat. No. P0018F, Beyotime Biotech.).

### 2.9. Statistical analysis

In this study, statistical analyses were performed using SPSS statistical software (version: 18.0, SPSS, Inc., Chicago, IL, USA). Data were defined as mean ± standard deviation (SD) and analyzed using one-way ANOVA followed by the Bonferroni test, when comparing differences between multiple groups. A p-value less than 0.05 was considered significant difference.

## 3. Results

### 3.1. GFP-expressing BMSCs were successfully cultured and identified

**Figure 1:**
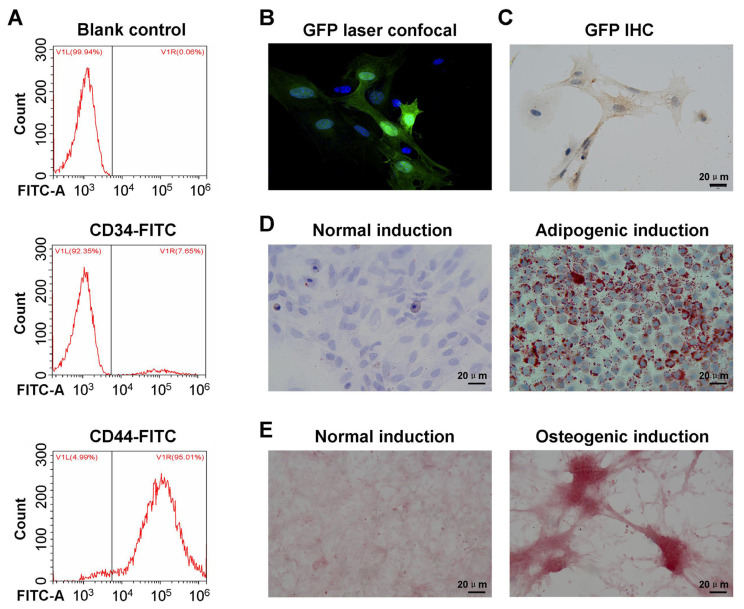
Identification and verification of the osteogenic/adipogenic differentiation ability of isolated BMSCs. A. Identification of isolated BMSCs with flow cytometry assay. B. Determination of the expression of the GFP protein in GFP-labeled BMSCs (GFP-BMSCs) using a laser confocal microscope. C. Verification of GFP expression in GFP-BMSCs using immunohistochemical assay. D. The adipogenic differentiation capacity of GFP-BMSCs was determined with alizarin-red staining. E. The osteogenic differentiation capacity of GFP-MBSCs was determined with oil-red O staining.

BMSCs were successfully infected/transduced with viral vectors expressing GFP (assigned as GFP-BMSCs). As shown in Figure 1A, the results showed that in BMSCs expressing GFP, the mean positive rate of cells expressing CD34-FITC was 7.65% and the mean positive rate of cells expressing CD44-FITC was 95.01%, indicating that the cells negatively expressed CD34 and positively expressed CD44, which meets the identification criteria of BMSCs. However, the Blank control group (as negative control group) was the control group without antibody incubation and there was no expression of CD34 and CD44 in BMSCs (Figure 1A). At the same time, combined with the results of morphological observation of the cells, it was confirmed that the isolated cells were BMSCs.

In this study, laser confocal analysis and immunohistochemical assay methods were used to evaluate the expression of GFP in BMSCs infected with lentivirus carrying the GFP gene (GFP-BMSCs). Laser confocal microscopy results (Figure 1B) showed that GFP was expressed in a large number of GFP-BMSCs, with a positive rate of more than 90%. Meanwhile, GFP was also expressed in the cell membrane of some GFP-BMSCs. Furthermore, the findings of the immunohistochemical assay (Figure 1C) indicated that a large number of GFP proteins were expressed in GFP-BMSCs.

### 3.2. GFP-BMSCs showed an obvious osteogenic and adipogenic differentiation ability

The oil-red O staining results showed that there was no obvious formation of lipid droplets in the normal induction of the GFP-BMSCs group (Figure 1D). However, in the adipogenic-induced GFP-BMSCs, round, orange-red, and fat droplets of different sizes appeared around the nucleus (Figure 1D). Meanwhile, the results of the alizarin red staining showed that there was no obvious formation of calcium nodules in GFP-BMSCs with normal induction (Figure 1E). However, in osteogenic-induced GFP-BMSCs, which showed obvious hyperchromatic calcified nodules (Figure 1E).

### 3.3. A bone marrow chimeric mouse model was successfully constructed

**Figure 2:**
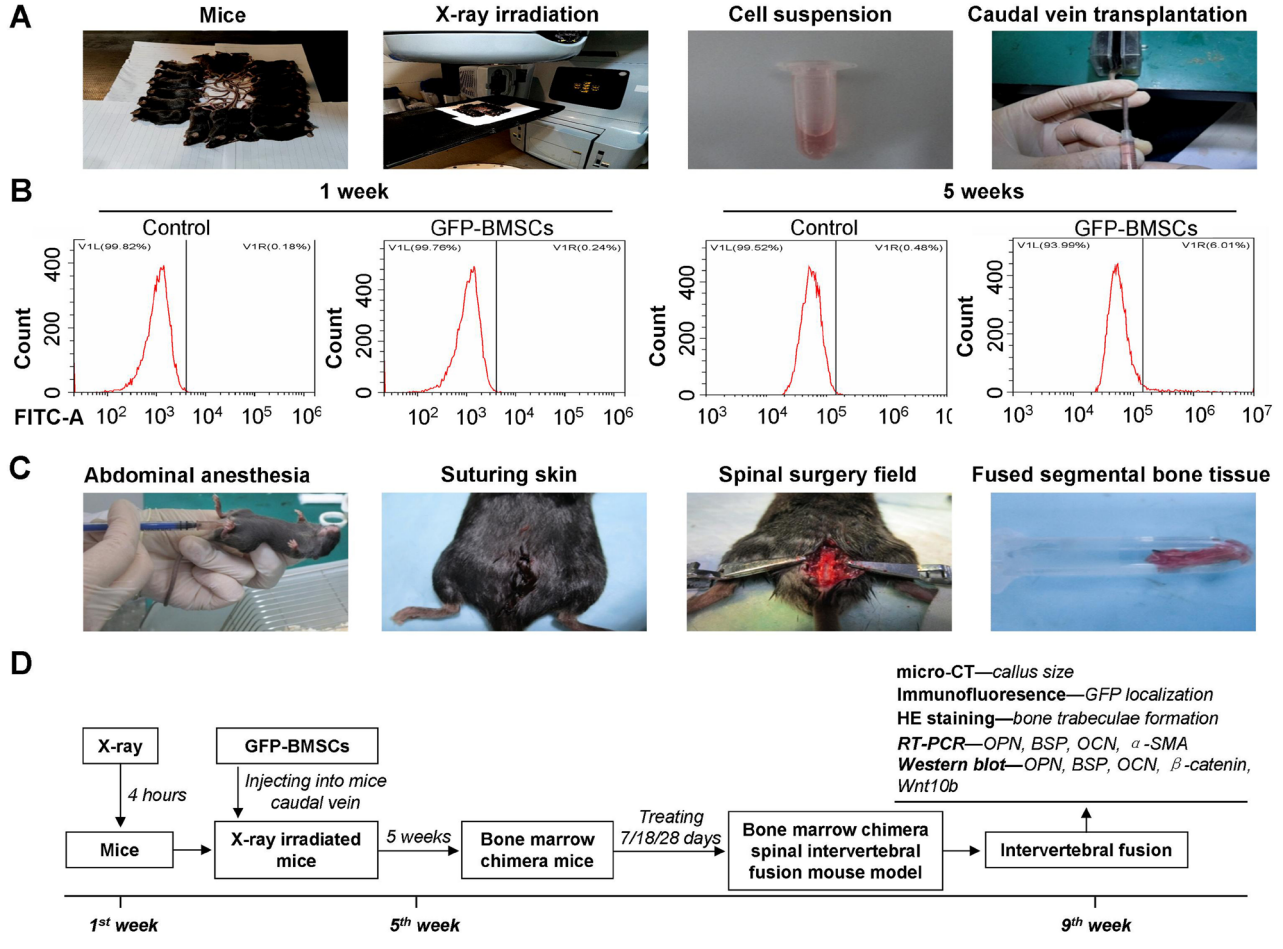
Establishment of the mouse model of the spinal intervertebral fusion of bone marrow chimera. A. Establishment of a bone marrow chimera model establishing process. The 6 Gy X-ray irradiated mice were transplanted with GFP-BMSCs to construct bone marrow chimeric mice. B. BMSCs extracted from mice in the GFP-BMSCs group were identified by flow cytometry assay. C. Establishment process of the bone marrow chimera spinal intervertebral fusion mouse model. D. Sketch of bone marrow chimera model and the bone marrow chimera spinal intervertebral fusion mouse model. The bone marrow chimera mice were treated as described in different groups and kept for 7, 18, and 28 days, to generate bone marrow chimera spinal intervertebral fusion mice. The intervertebral fused bone was then isolated for the following experiments.

Mice waiting for GFP-BMSCs transplantation in the caudal vein were irradiated with 6 Gy X-ray (Figure 2A) and then kept for 1 week. The prepared GFP-BMSCs were transplanted into the tail vein of X-ray-irradiated mice (GFP-BMSCs group) to construct bone marrow chimeric mice (Figure 2A), while cell-free medium was injected into the tail vein of X-ray-irradiated mice, assigned as Control group. BMSCs in mice from the GFP-BMSCs group and mice from the Control group were extracted at the first week and the fifth week after transplantation for flow cytometry detection. The results showed that there were no GFP expressions in BMSCs extracted from mice in both the Control group (0.18%) and the GFP-BMSCs group (0.24%) in the 1st week. BMSCs extracted from mice in the GFP-BMSCs group expressed a higher level of GFP (6.01%); however, the level of GFP in BMSCs extracted from mice in the Control group was lower (0.48%) in the 5th week (Figure 2B). Therefore, the GFP-expressing BMSCs (GFP-BMSCs) in vitro were successfully transplanted into the X-ray-irradiated recipient mice and GFP was stably expressed in vivo, indicating that bone marrow chimeric mice were successfully constructed.

### 3.4. SDF-1 increased callus size at the intervertebral bone graft fusion site

**Figure 3:**
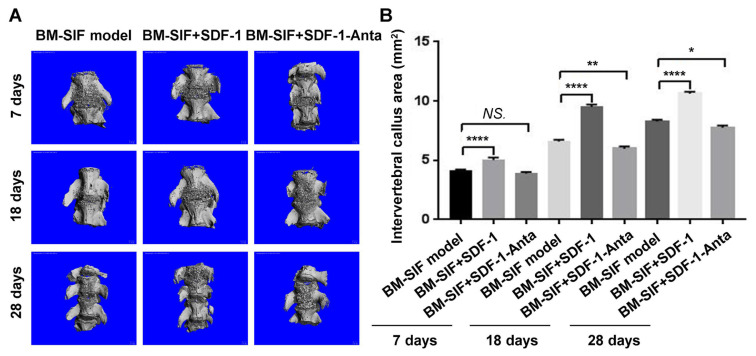
Evaluation of callus size at the intervertebral bone graft fusion site of the bone marrow chimera of mice. A. The size of the callus at the intervertebral bone graft fusion site was evaluated using micro-CT and three-dimensional imaging, 7, 18, and 28 days after surgery. B. Statistical analysis of the callus size of mice of different groups. The statistical differences (significant values) are illustrated in the images. ^*^p < 0.05, ^**^p < 0.01, and ^****^p < 0.0001 represented the significant differences. NS: no significance.

The bone marrow chimera spinal intervertebral fusion mouse model was successfully established as shown in Figure 2C. In this study, microCT observation and three-dimensional imaging were performed on the callus at the intervertebral bone graft fusion site of mice from the BM-SIF model group, the BM-SIF+SDF-1 group, and the BM-SIF+SDF-1-Anta group (Figure 3A). Analysis of the callus area showed that SDF-1 treatment (BM-SIF+SDF-1 group) significantly increased the callus area of mice compared to the BM-SIF model group, 7, 18, and 28 days after operation (Figure 3B, all p < 0.05). The callus area of mice in the treated BM-SIF+SDF-1-Anta group was significantly lower compared to mice in the BM-SIF+SDF-1 group (Figure 3B, all p < 0.05). At 7 days after operation, there was no significant difference in callus area between mice in the BM-SIF model group and the BM-SIF+SDF-1-Anta group (Figure 3B, p > 0.05). At 18 and 28 days after operation, the callus area of the mice in the BM-SIF model group was higher than that of the mice in the BM-SIF+SDF-1-Anta group (Figure 3B, both p < 0.05).

### 3.5. SDF-1 promoted the formation of bone trabeculae in the callus at the intervertebral bone graft fusion site

**Figure 4:**
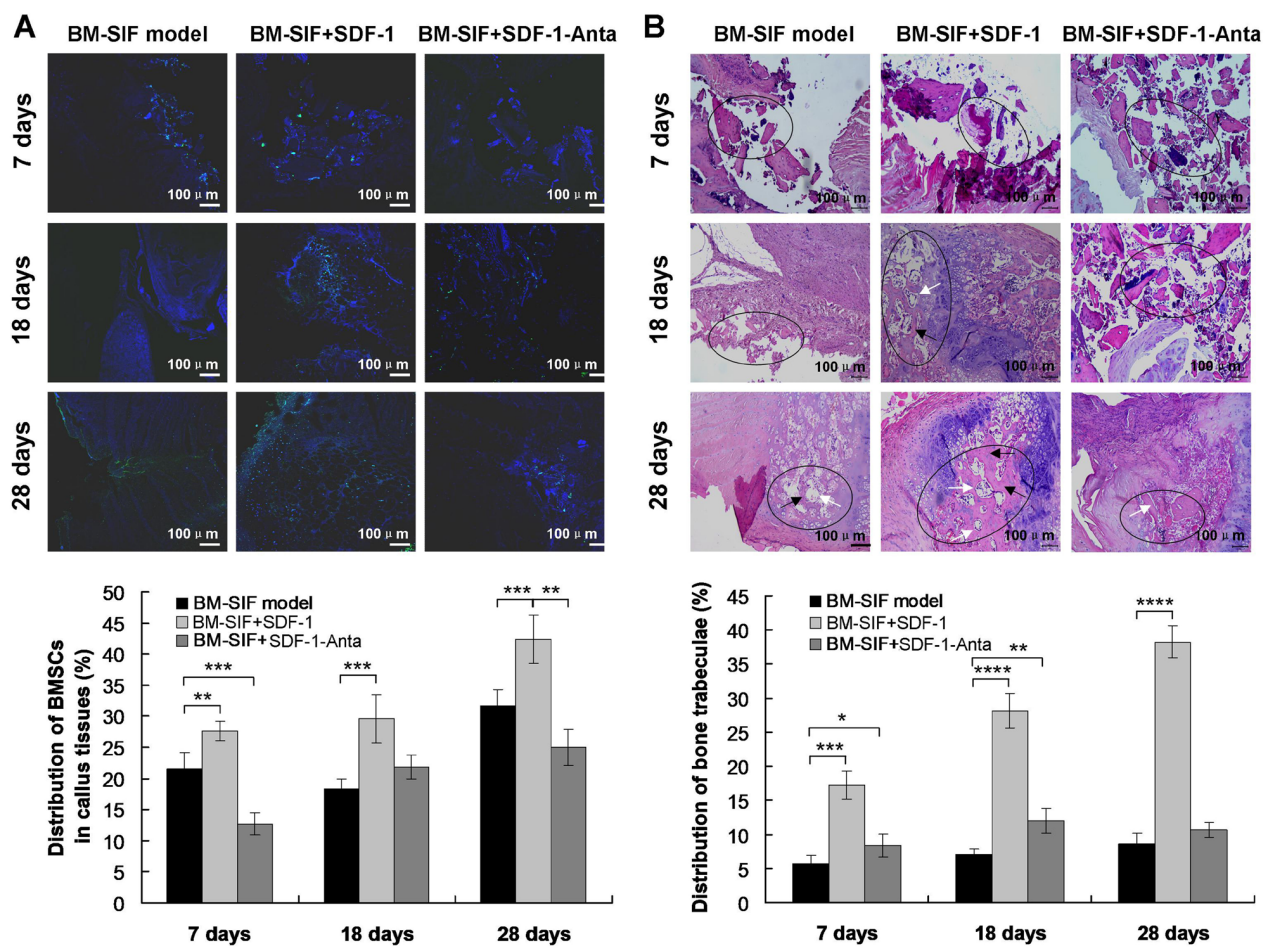
Detection of the distribution of BMSCs and the formation of bone trabeculae in the callus at the intervertebral bone graft fusion site of mice from different groups. A. The distribution of BMSCs in callus tissues was determined using an immunofluoresence assay, 7, 18, and 28 days after surgery. B. The formation of bone trabeculae in the callus was determined by HE staining, 7, 18, and 28 days after surgery. The scale bars are illustrated in the images. ^*^p < 0.05, ^**^p < 0.01, ^***^p < 0.001 and ^****^p < 0.0001 represented significant differences. The white arrows represented the osteoblasts and black arrows represented the bone trabeculae. The black circle labeled the areas involving osteoblasts and bone trabeculae.

The number of green fluorescent BMSCs (GFP-BMSCs) at the intervertebral bone graft fusion site was detected by a laser confocal microscope (Figure 4A). The results showed that the number of GFP-positive BMSCs (GFP-BMSCs) in mice from the BM-SIF+SDF-1 group was significantly higher than in mice from the BM-SIF model group at 7 days (p < 0.05), 18 days (p < 0.05), and 28 days (p < 0.05) (Figure 4A). Therefore, BMSCs were widely distributed in the callus of the intervertebral bone graft fusion site.

On the 7th, 18th, and 28th days after successful construction of intervertebral fusion mouse model, the mice were killed, and callus tissues of intervertebral fusion site were stained with HE staining. HE staining results showed that on the 7th day after surgery, blood scabs were formed, and the tissue structures were disordered in all three groups. More fibroblasts could be seen in the callus tissue of mice from the BM-SIF+SDF-1 group (Figure 4B), a small number of fibroblasts or no cartilage formation can be seen in mice from the BM-SIF model group and the BM-SIF-SDF-1-Anta group (Figure 4B). On the 18th day after operation, more chondrocytes and fibroblasts were seen in callus of mice from the BM-SIF model group (Figure 4B). In the BM-SIF+SDF-1 group, there was a large amount of cartilage formation and a small amount of fibrous tissue around the periphery, gradually forming transitional bone trabeculae (Figure 4B). There were a few chondrocytes and fibroblasts in the callus of the BM-SIF+SDF-1-Anta group, and there was almost no new bone (Figure 4B). The histological process of callus in the BM-SIF+SDF-1 group was significantly faster than that in the other two groups. On the 28th day after the operation, the residual bone trabeculae in the callus of the BM-SIF model group were almost completely absorbed, a large amount of cartilage was formed, and a small amount of new bone trabeculae was formed (Figure 4B). Hundreds of new trabecular bone tissue can be seen in the callus of mice in the BM-SIF+SDF-1 group, and the trabeculae were dense and fused into blocks (Figure 4B). The percentage of new bone trabeculae in mice in the BM-SIF+SDF-1 group was significantly higher than in the BM-SIF model group at 7 days (p < 0.05), 18 days (p < 0.05), and 28 days (p < 0.05) (Figure 4B). The histological process of callus in mice from the BM-SIF+SDF-1-Anta group was obviously slower than that of the other two groups (Figure 4B).

### 3.6. SDF-1 upregulated the transcription and expression of osteogenic molecules in the callus at the intervertebral bone graft fusion site

**Figure 5:**
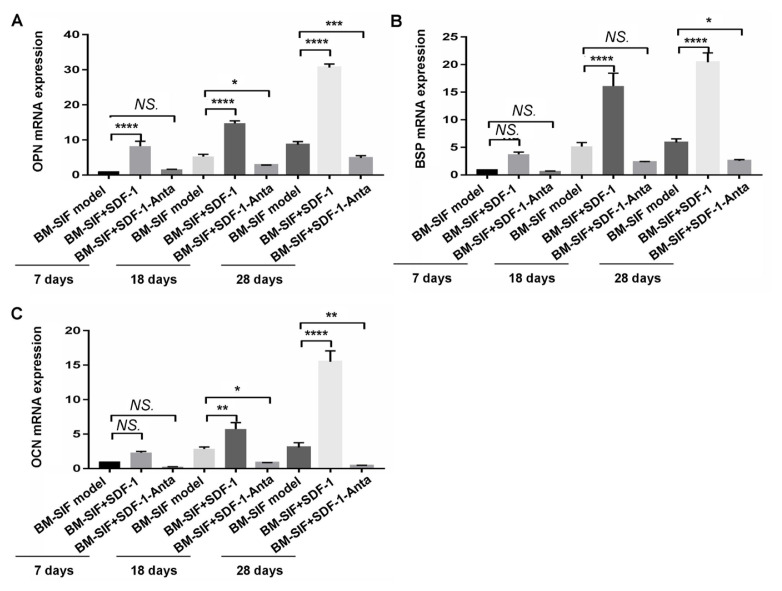
Effects of SDF-1 treatment on the expression of osteogenic gene transcription in callus at the intervertebral bone graft fusion site of mice, determined by the RT-PCR assay. The transcription of the OPN gene (A), the BSP gene (B), and the OCN gene (C) in the callus of mice was analyzed and compared between different groups, 7, 18, and 28 days after surgery. The statistical differences (significant values) are illustrated in the images. ^*^p < 0.05, ^**^p < 0.01, ^***^p < 0.001, and ^****^p < 0.0001 represented significant differences. NS: no significance. Number of samples: n = 12 for BM-SIF model group, BM-SIF+SDF-1 group, and BM-SIF+SDF-1-Anta group.

The transcription of the OPN gene in mice in the BM-SIF+SDF-1 group was significantly higher than in mice in the BM-SIF model group and the BM-SIF+SDF-1-Anta group, 7 days, 18 days, and 28 days after surgery (Figure 5A, all p < 0.05). At 18 and 28 days, the transcription of the OPN gene in the BM-SIF model group was higher compared to that in the BM-SIF+SDF-1-Anta group (Figure 5, all p < 0.05). The transcription of the BSP gene in mice in the BM-SIF+SDF-1 group was higher compared to mice in the BM-SIF model group and the BM-SIF+SDF-1-Anta group at 18 days and 28 days after surgery (Figure 5B, all p < 0.05). The transcription of the BSP gene in mice from the BM-SIF model group was higher than that of mice from the BM-SIF+SDF-1-Anta group 28 days after surgery (Figure 5B, all p < 0.05). At 18 days and 28 days after surgery, the transcription of the OCN gene in mice from the BM-SIF+SDF-1 group was higher compared to mice from the BM-SIF model group and the BM-SIF+SDF-1-Anta group (Figure 5C, p < 0.05), and the transcription of the OCN gene in mice from the BM-SIF model group was higher than in mice from the BM-SIF+SDF-1-Anta group (Figure 5C, p < 0.05).

**Figure 6:**
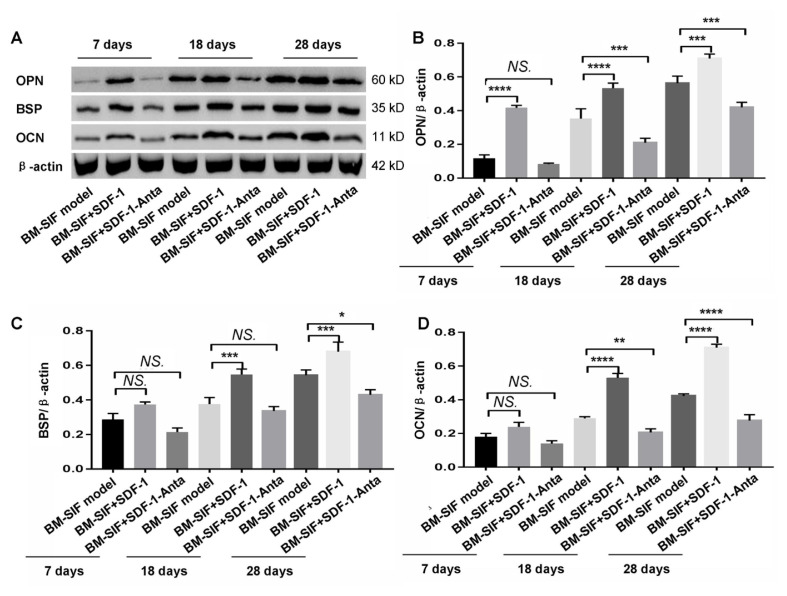
Treatment with SDF-1 increased the expression of osteogenic molecules in the callus at the intervertebral bone graft fusion site of mice. A. Western blot images of osteogenic molecules expression in the callus of mice, 7, 18, and 28 days after surgery. The expressions of OPN (B), BSP (C), and OCN (D) in the callus of mice were analyzed and compared between different groups. The statistical differences (significant values) are illustrated in the images. ^*^p < 0.05, ^**^p < 0.01, ^***^p < 0.001, and ^****^p < 0.0001 represented significant differences. NS: not significant. Number of samples: n = 12 for BM-SIF model group, BM-SIF+SDF-1 group, and BM-SIF+SDF-1-Anta group.

The results of the Western blot assay (Figure 6A) for osteogenic gene expression were equal to the trend of gene transcription change from the RT-PCR findings. Briefly, SDF-1 treatment significantly increased the expression of OPN (Figure 6B), BSP (Figure 6C), and OCN (Figure 6D) in the callus of mice compared to those of mice from the BM-SIF model group (all p < 0.05). While the expression of OPN (Figure 6B), BSP (Figure 6C), and OCN (Figure 6D) in the callus of mice from the BM-SIF+SDF-1-Anta group was markedly lower compared to those of mice from the BM-SIF+SDF-1 group (all p < 0.05).

### 3.7. SDF-1 induced fibroblast proliferation in the callus at the intervertebral bone graft fusion site

**Figure 7:**
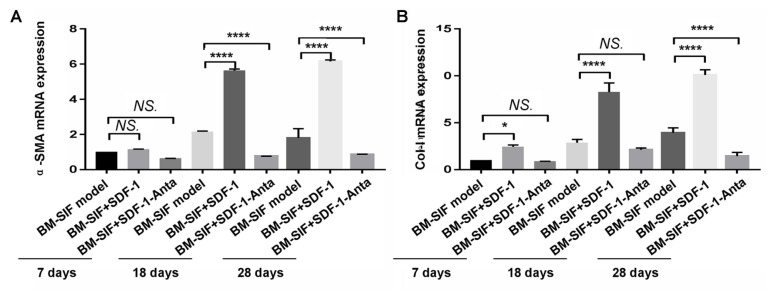
Treatment with SDF-1–induced fibroblast proliferation in the callus at the intervertebral bone graft fusion site of mice, according to the RT-PCR assays. A. Statistical analysis of the transcription of the fibroblast marker, α-SMA gene, between different groups. B. Statistical analysis of the fibroblast marker, Col-1 gene transcription, between different groups. The statistical differences (significant values) are illustrated in the images. ^*^p < 0.05, and ^****^p < 0.0001 represented significant differences. NS: not significant. Number of samples: n = 12 for BM-SIF model group, BM-SIF+SDF-1 group, and BM-SIF+SDF-1-Anta group.

In this study, we used the RT-PCR assay to detect transcriptions of fibroblast markers, including α-SMA gene and the Col-1 gene, in the callus at the intervertebral bone graft fusion site. The results showed that the transcription of the α-SMA gene in the callus of mice from the BMSIF+SDF-1 group was significantly higher compared to mice from the BM control group and the BM-SIF+SDF-1-Anta group (Figure 7A, all p < 0.05), while the transcription of the α-SMA gene in the callus of mice from the BM-SIF model group was significantly higher than that of mice from the BM-SIF+SDF-1 group (Figure 7A, p < 0.05), at 18 and 28 days after surgery. At 7 days, 18 days, and 28 days after surgery, Col-1 gene transcription in the callus of mice from the BM-SIF+SDF-1 group was significantly higher compared to that in the callus of mice from the BM-SIF model group and the BM-SIF+SDF-1-Anta group (Figure 7B, all p < 0.05). Meanwhile, the transcription of the Col-1 gene in the callus of mice in the BM-SIF model group was significantly higher compared to mice in the BM-SIF+SDF-1-Anta group (Figure 7B, p < 0.05).

### 3.8. SDF-1 modulated Wnt/β-catenin pathway by increasing the expression of Wnt10b and β-catenin in the callus at the intervertebral bone graft fusion site

**Figure 8:**
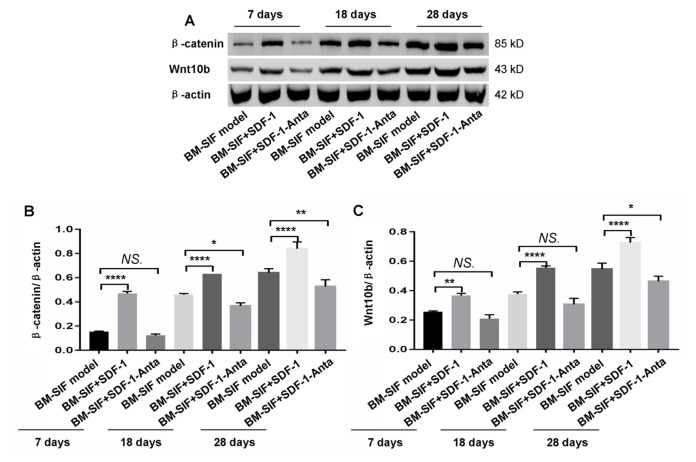
Treatment with SDF-1 increased the expression of Wnt10b and β-catenin expression in the callus at the intervertebral bone graft fusion site of mice. A. Western blot assay of Wnt10b and β-catenin expression in the callus of mice, 7, 18, and 28 days after surgery. The β-catenin (B) and Wnt10b (C) expressions in the callus of mice were analyzed and compared between different groups. The statistical differences (significant values) are illustrated in the images. ^*^p < 0.05, ^**^p < 0.01, and ^****^p < 0.0001 represented significant differences. NS: not significant. Number of samples: n = 12 for BM-SIF model group, BM-SIF+SDF-1 group, and BM-SIF+SDF-1-Anta group.

A Wnt/β-catenin signaling pathway key molecule, β-catenin, and bone formation associated molecule, Wnt10b, were verified in the callus at the intervertebral bone graft fusion site using a Western blot assay (Figure 8A). The results showed that β-catenin (Figure 8B) and Wnt10b (Figure 8C) gene transcriptions were significantly higher in the callus of mice in the BM-SIF+SDF-1 group compared to mice in the BM-SIF model group, 7 days, 18 days, and 28 days after surgery (all p < 0.05). However, the β-catenin gene transcriptions (Figure 8B) were markedly reduced in the callus of mice in the BM-SIF+SDF-1-Anta group at 18 days and 28 days after surgery, and the transcriptions of the Wnt10b gene (Figure 8C) were markedly decreased in mice in the BM-SIF+SDF-1-Anta group at 28 days after surgery, compared to those of mice in the BM-SIF model group (all p < 0.05).

## 4. Discussion

Lumbar disc degenerative disease is progressive irreversible damage to nerve roots and chronic lumbar pain and is one of the most common causes of spinal degeneration (Yang et al., 2022). Lumbar disc degenerative disease is affected by a variety of risk factors (Ye et al., 2021), such as weight bearing, autoimmune diseases, nutritional imbalance, molecular and cellular abnormalities. The commonly used clinical treatment methods for degenerative disc lumbar disease are conservative physical therapy, drug analgesia, etc. (Hayden et al., 2021), but spinal fusion technology is the most commonly used surgical method, which can promote bone fusion and spinal biomechanical reconstruction. Therefore, spinal fusion technology has been recognized as the “gold standard” of spinal surgery. However, the failure of spinal fusion is a problem that spinal surgeons must face in clinical work. Spinal fusion surgery may involve the use of autologous or allogeneic bone tissue and, in some cases, the addition of bone substitutes. The fusion rate for autotransplantation of the iliac bone is higher than 90%, but this surgical method will cause complications (Hofmann et al., 2020). Currently, the mechanism of bone graft fusion in spinal fusion has not been fully clarified. Therefore, the effective improvement of the fusion rate of spinal fusion at the molecular level has become an important problem to solve in the field of spinal surgery.

Intervertebral fusion is a very complicated molecular biological process. In the whole process of intervertebral fusion, the final differentiation of bone grafts is mainly completed by bone conduction and bone induction (Zhang et al., 2019; Lu et al., 2020). With the digestion and absorption of transplanted bone, with the help of regenerated capillaries, bone-inducing factors in the residual culture matrix and host mesenchymal stem cells, mesenchymal stem cells change into osteoblasts and then become new bone under induction of bone-inducing factors.

In recent years, cell therapy has been widely used in the treatment of various diseases. Mesenchymal stem cells (MSCs) can differentiate into other functional cells in a specific environment, such as bone cells that could be used in the treatment of orthopedic diseases (Yang et al., 2021). BMSCs include mainly endogenous BMSCs and exogenous BMSCs. Exogenous BMSCs have obvious defects (Dong et al., 2019), while endogenous BMSCs have no immune rejection and can continuously transfer to the injury site and participate in injury repair for a period of time after injury (Zhai et al., 2018). Autologous BMSCs have the ability to spontaneously migrate to the injured site and participate in the repair of the corresponding tissues (Shen et al., 2016). However, without interference factors, only a small number of BMSCs participate in bone repair and their effect on bone repair after bone trauma or large bone fracture is limited. However, it has not been reported whether BMSCs can increase osteogenic differentiation by increasing chemokines to promote the movement of BMSCs to the intervertebral fusion site.

A previous study (Yang et al., 2018) has shown that there is a high expression of SDF-1 at the site of bone tissue repair. Meng et al. (2021) reported that the SDF-1/CXCR4 signaling pathway can promote the collection of somatic cells and related factors necessary for bone healing to the site of bone damage and participate in the bone regeneration process at the site of bone damage. A recent study (Wang et al., 2017) has also found that MSC migration is mediated by the SDF-1/CXCR4 axis, and SDF-1 can reach the site of bone injury for osteogenic repair (Wang et al., 2018). The SDF-1/CXCR4 signaling pathway can cause knee cartilage degeneration and capillary formation in osteoarthritis (OA) (Qin et al., 2019). Because OA cartilage tissue is similar to the microenvironment after intervertebral space bone grafting, such as high internal stress, low blood supply, hypoxia, and low nutrients, this study inferred that the SDF-1/CXCR4 signaling pathway also played an important role in spinal interbody bone grafting and fusion. In this study, spinal intervertebral fusion mice of the bone marrow chimera were successfully constructed, which were characterized by the green fluorescent label of BMSCs in mice and used to track and observe whether SDF-1 could migrate to the intervertebral fusion site. Meanwhile, this study used this model to explore the role of SDF-1 in promoting fruit healing, endogenous BMSCs targeted chemotaxis, and bone repair.

Fusion of the SDF-1 and G protein coupled reaction protein kinase can induce transmembrane spiral changes of CXCR4 and CXCR7 and then activate the pi3k/akt pathway in the cytoplasm (Ma et al., 2019). Phosphatidylinositol 3-kinase (PI3Ks), as the main downstream molecule of the tyrosine kinase and the G protein coupled receptor, can catalyze the production of phosphatidylinositol 3,4,5-triphosphate (PIP3) and the second messenger, and activates downstream molecules protein kinase B (PKB/AKT) and glycogen synthase kinase-3 β (GSK-3 β). Therefore, PKB/AKT/GSK-3 β signaling pathway transmits a variety of biological signals to cells and then regulates the processes of cell proliferation, differentiation, and apoptosis (Liang et al., 2015). A previous study (He et al., 2020) has shown that the maturation and mineralization of rat osteoblasts are related to PI3K/AKT/GSK3β/β-catenin signal pathway. Meanwhile, wnt/β-catenin signaling pathway plays a key role in embryonic stem cell growth, development and differentiation, bone production, and insulin glargine metabolism (Lei et al., 2020). The Wnt pathway can regulate osteoblast differentiation, bone matrix production, and calcification, and endanger osteoblast production and bone resorption (Hong et al., 2019). Zhang et al. (2015) found that in the Wnt signaling pathway, Wnt10b is the canonical expression of the wnt ligand in bone formation, which is unique to the activity of BMSCs. The knockout of the Wnt10b gene caused bone loss and a progressive reduction in BMSCs in the elderly (Li et al., 2021); thus, Wnt/β-catenin signaling pathway is closely related to bone production. Wnt/β-catenin signaling pathway acts on a series of whole bone production processes, including the production, differentiation, and reproduction of BMSCs in osteoblasts. However, it has not been reported whether SDF-1 also regulates the formation of new bone through this signal pathway. Previous results show that SDF-1 can increase osteogenesis at the intervertebral fusion site. To verify whether SDF-1 regulates osteogenesis by activating the Wnt/β-catenin signaling pathway, we used a Western blot assay to determine the expression of β-catenin, Wnt10b, BSP, OCN, and OPN, and used an RT-PCR assay to determine the transcription of Col-1, α-SMA, Wnt10b, BSP, OCN, OPN gene. The results showed that the osteogenesis-related indices of the mice in the BM-SIF+SDF-1 group were higher than those of other groups, and the expression of key proteins (β-catenin and Wnt10b) of the mice in the BM-SIF+SDF-1 group were significantly higher than those of the mice in the other two groups. Therefore, SDF-1 can promote the directional chemotaxis of BMSCs and osteogenesis by regulating the Wnt/β-catenin signaling pathway.

There were also some deficiencies in this study. Firstly, this study had only been verified in mice, and more research was needed for other animals and the application of the transformation of subsequent achievements in clinical practice. Secondly, the classic Wnt/β-catenin signaling pathway had been selected in this study; however, it was not known whether the other signaling pathways involved in osteogenesis and playing a role in the regulation of stem cell osteogenesis needed further verification. Thirdly, only the callus area of the intervertebral fusion part of the callus was detected in this study in mice. Although it was scientific, micro-CT analysis usually used to evaluate the bone volume fraction (BVF), bone mineral content (BMC), bone mineral density (BMD), and trabecular bone analysis was used to evaluate the fusion effect. Fourthly, according to the present data, there may be a clear association between SDF-1 treatment and Wnt/β-catenin pathway in the callus. However, it remains unclear whether SDF-1 really causes the migration of BMSCs or the responses to tissue injury repair by the Wnt/β-catenin pathway. In the following study, we would verify if this is a cause or consequence of migration or repair process, or both. Finally, xenotransplantation and chemotaxis seem to be an important part of the present study; however, it has not been sufficiently investigated in this study.

## 5. Conclusion

In summary, this study used bone marrow chimeric mice with GFP-BMSCs to construct a mouse model of spinal intervertebral fusion. When there was a bone graft area in the spine, the BMSCs will theoretically migrate to the bone graft fusion site and differentiate into osteoblasts, eventually inducing bone formation. To verify the induction of SDF-1 in the directional migration of BMSCs, we added SDF-1 locally to the bone graft fusion site when building the bone marrow chimera spinal intervertebral fusion mouse model. MicroCT was used to detect the callus area of each group of mice at different time points, and a laser confocal microscope was used to detect the directional chemotaxis of GFP-BMSCs to assess the osteogenic effect of SDF-1 on the spinal intervertebral fusion. In vivo and in vitro experiments showed that SDF-1 could induce BMSCs to chemotaxis to the intervertebral fusion site and promote osteogenic differentiation in the successfully constructed mouse model. SDF-1 might participate in the formation of new bone at the intervertebral fusion site in spinal intervertebral fusion mice of the bone marrow chimera by regulating Wnt/β-catenin signaling pathway and modulating BMSCs proliferation. This study would further clarify the mechanism of SDF-1 and provide a promising treatment idea for spinal intervertebral fusion. The SDF-1–associated molecules could become novel products in the near future, which can be used in the clinic to improve spinal intervertebral fusion through the transformation of technical achievements.

## Figures and Tables

**Table:** The RT-PCR primers of the targeting genes.

Genes	Sequences (5′-3′)	Length (bp)
GAPDH-Forward	AGAGTGTTTCCTCGTCCCGTAG	204
GAPDH-Reverse	CTTGACTGTGCCGTTGAATTTG	
Col-1-Foward	GGAGAGAGTGCCAACTCCAG	183
Col-1-Reverse	GTGCTTTGGAAAATGGTGCT	
α-SMA-Forward	CCGAGATCTCACCGACTACC	120
α-SMA-Reverse	TCCAGAGCGACATAGCACAG	
BSP-Forward	AGAATCTCCTTGCGCCACA	118
BSP-Reverse	TCGTCATCATCGTCGTCCA	
OCN-Forward	CCTGGAGATTATATTTGCCGTA	107
OCN-Reverse	AAGGCATACTGAGTCACACC	
OPN-Forward	AGAATCTCCTTGCGCCACA	118
OPN-Reverse	TCGTCATCATCGTCGTCCA	
